# A new account of the conditioning bias to out-groups

**DOI:** 10.3389/fpsyg.2015.00197

**Published:** 2015-02-24

**Authors:** Junhua Dang, Shanshan Xiao, Lihua Mao

**Affiliations:** ^1^Department of Psychology, Lund UniversityLund, Sweden; ^2^Department of Psychology, Peking UniversityBeijing, China

**Keywords:** conditioning bias, in-group favoritism, out-group derogation, prepared learning, latent inhibition

## The conditioning bias to out-groups

In classical fear conditioning, a neutral stimulus, once paired with an aversive event for several times, can induce fear reaction by itself. Compared with fear-irrelevant stimuli such as birds and butterflies, fear-relevant stimuli such as spiders and snakes are more readily associated with aversive events. This prepared learning phenomenon is highly resistant to extinction, insensitive to cognitive manipulations, and could be acquired in only one trial (Seligman, 1971; Öhman and Mineka, 2001). From an evolutionary perspective, human beings and non-human primates are predisposed to learn to fear spiders and snakes because such preparedness conferred a selective advantage to our ancestors over conspecifics that were not prepared.

Inspired by recent studies that showed overlapping neural systems underlying racial bias and fear conditioning (Phelps et al., 2001; Olsson and Phelps, 2004; Olsson et al., 2005), tested whether prepared learning can be extended to a socio-cultural context. They employed the differential conditioning paradigm that consisted of three phases—habituation, acquisition, and extinction—presented in sequence (Öhman and Mineka, 2001). The conditioned stimuli (CS) were two pictures of Black individuals and two pictures of White individuals. During habituation, all pictures were presented without reinforcement. During acquisition, one of the pictures from each race (CS+) was accompanied by an electric shock (the unconditioned stimulus, US), whereas the other (CS-) was not. During extinction, no shocks were administered. The conditioned fear response (CR) was obtained by subtracting participants' skin conductance responses (SCRs) to the CS- from their SCRs to the CS+ for each race. The main result was that the CR to racial out-group faces persisted whereas the CR to in-group faces was fully extinguished in the absence of the US.

## Available explanations and their shortages

The finding for racial faces resembles the pattern observed for spiders and snakes, indicating that racial out-group faces may serve as a signal of social threat and be processed in a manner similar to fear-relevant stimuli. However, it may be too early to conclude that the conditioning bias to out-groups could be considered as a typical prepared learning phenomenon. Theoretically, the evidence that human populations differentiated into different races recently in evolutionary history because of geographic isolation of different lineages makes it unlikely that humans could have evolved mechanisms specifically to learn to fear different races, since being genetically prepared to learn to fear other races could not provide any selective advantage (Maia, 2009). In the meantime, to be regarded as an instance of prepared learning, an association has to fulfill not only the criterion of resistance to extinction, but also the criteria of one trial learning and insensitivity to cognitive manipulations. However, researchers have found that the conditioning bias to out-groups was abolished by cognitive manipulations. During the extinction phase, such bias disappeared once the experimenter informed participant that no more electric shocks would be presented and the shock electrode was removed from participants' arm (Mallan et al., 2009).

Recently, a theoretical analysis argued that the conditioning bias to out-groups could be explained by standard learning theory (Maia, 2009). Specifically, the author suggested that the conditioning bias to out-groups might be mediated by a mechanism called latent inhibition. That is to say, a familiar but unassociated stimulus takes longer to acquire meaning (as a signal) than a new stimulus. In the intergroup context, people are generally familiar with their in-groups, so they need more time to associate their in-groups with the unconditioned stimuli. During the same acquisition time, this association would not become so strong as the association between the unfamiliar out-groups and the unconditioned stimuli. As a result, it would also be easier to extinguish. The pattern of Olsson et al.'s (2005) findings was indeed consistent with this account. Besides the finding that the CR to out-groups remained significant after extinction, the CR to out-groups also appeared to be stronger than the CR to in-groups during acquisition, although no explicit comparison was done in Olsson et al.'s (2005) study.

In our opinion, although it is not appropriate to consider the conditioning bias to out-groups as preparedness and use the evolutionary account to explain it, the latent inhibition view does not provide a convictive explanation, either. For example, a very recent research using the minimal group paradigm found that after arbitrarily assigned to different groups according to a trivial criterion (e.g., perceptual style), participants more readily learned a fear response when an aversive stimulus was paired with out-group faces than when it was paired with in-group faces (Navarrete et al., 2012). That is to say, the CR to out-groups was stronger than the CR to in-groups during acquisition. However, unlike Olsson et al.'s (2005) results, the CRs to both in-groups and out-groups were fully extinguished during the extinction phase. Latent inhibition could not explain why the CR to out-groups were stronger than the CR to in-groups in that the two groups were both formed in the lab, having little difference in their familiarity, and thus should not result in different learning rate.

Indeed, Olsson et al. (2005) also questioned the evolutionary account but instead argued that sociocultural learning about the identity and qualities of out-groups provided the basis for persistence of the CR to out-groups. That is to say, repeated exposure to negative information about out-groups might prepare people to fear newly encountered out-group members. Similar idea has also been advanced by other researchers very recently (Mallan et al., 2013). This account seems evolutionarily more plausible and has a potential to reconcile the extinguished CR to out-groups in the minimal group paradigm (Navarrete et al., 2012). However, these researchers did not elaborate the specific underlying mechanism. In the meanwhile, this account still has a limitation to explain the reduced CR to in-groups during acquisition (Olsson et al., 2005; Navarrete et al., 2012).

## A new account

We think in order to provide a more complete explanation, we have to take into account the theories and findings in the area of intergroup relationship. Traditionally, research in this field appeared to accept, at least implicitly, the idea that relationships between in-groups and out-groups were characterized by antagonism, conflict, and mutual contempt. As a result, the favoritism toward in-groups and the derogation toward out-groups were studied interchangeably, as if they were two sides of the same coin. Recently, more and more researchers began to re-consider this issue and suggested in-group favoritism and out-group derogation were separable phenomena (e.g., Brewer, 1999; Hewstone et al., 2002). For example, most minimal group studies rating the in-group and the out-group separately found that categorization into groups led to positive in-group ratings in the absence of negative out-group ratings (Brewer, 1979). Further, the positive in-group bias found in the allocation of positive resources (e.g., money) using the minimal group paradigm disappeared when negative outcomes (e.g., noise) were distributed (e.g., Mummendey et al., 1992), suggesting humans are willing to differentially benefit the in-groups rather than harm out-groups. Recent research in developmental psychology (e.g., Aboud, 2003) and social neuroscience (e.g., Van Bavel et al., 2011) provided further supporting evidence for this perspective. Indeed, researchers even found that group formation and positive in-group regard had intragroup origins and did not require comparison with a contrasting out-group (Gaertner et al., 2006).

According to this new perspective, in-group favoritism arises from a self-anchoring mechanism such that self-evaluation could automatically extend to in-groups. As recent research revealed, personal trait self-esteem is positively correlated with in-group favoritism and this association holds not only when the in-group and the out-group are equivalent (i.e., classed only by a trivial criteria) but also when the in-group is objectively less favorable than the out-group (Gramzow and Gaertner, 2005). Because people generally have a good view of themselves, they would automatically transfer such positivity to the perception regarding their in-groups. For example, using a typical paradigm measuring spontaneous trait inferences in a minimal group setting, Otten and Moskowitz (2000) demonstrated that behaviors that implied positive traits about an in-group member were more likely to be categorized in a manner consistent with the implied trait. However, there was no facilitation of trait inference to out-group members performing negative behaviors, suggesting there was evidence for implicit in-group favoritism but not out-group derogation.

In contrast, out-group derogation occurs when the very existence of the out-group, or its goals and values, are seen as a threat to the maintenance and the social identity of the in-group (Brewer, 2007). The integrated threat theory (Stephan and Stephan, 2000) distinguishes four different sources of experienced threat from a specific out-group: realistic threat (threat to in-group physical and psychological well-being), symbolic threat (threat to the stability of in-group values and beliefs), intergroup anxiety (personal discomfort arising from actual or anticipated interactions with out-groups), and negative stereotypes (beliefs about out-group characteristics implying unfavorable interactions and negative consequences). Following studies supported this model by showing that these threat perceptions (especially the first three perceptions) significantly predicted negative attitudes toward out-groups and mediated the effects of other predictors such as intergroup contact (Stephan et al., 2002).

Thus, the conditioning bias to out-groups may relate to the mechanism underlying in-group favoritism and out-group derogation. On one hand, since individuals tend to automatically transfer the positive view of themselves to their in-groups, either novel ones (e.g., formed arbitrarily in a lab) or real ones (e.g., Blacks or Whites), their in-groups would be rated more positively than their out-groups. When in-group faces and out-group faces serve as CS, in-group faces, initially positive stimuli, would need more time to acquire an aversive meaning (Zanna et al., 1970). Consequently, the CR to in-group faces would be weaker than the CR to out-group faces during the acquisition phase. Such inferences are indeed consistent with both Olsson et al.'s (2005) findings (using racial groups) and Navarrete et al.'s (2012) findings (using minimal group paradigm).

On the other hand, out-groups would generally be rated neutral if the out-group does not pose a threat to the in-group. Previous research revealed that the CR to fear-irrelevant neutral stimuli could be extinguished during the extinction phase (Seligman, 1971). That is also what Navarrete et al. (2012) found in a minimal group setting. However, under certain conditions (e.g., when high conflict between groups occurs), out-groups would be rated negatively because they are perceived as a threat to in-groups. In Olsson et al.'s (2005) study, they used two racial groups, Whites and Blacks. Obviously, these two racial groups shared a history of discrimination, prejudice, and even war. The racial issue is still sensitive and tense in today's America and both groups hold a negative attitude toward each other. Since fear responses to CS that already have a negative valence are easier to acquire (Zanna et al., 1970) and thus harder to extinguish, there is no wonder that these authors found the CR to out-groups failed to be extinguished. Although this analysis shares some features with the sociocultural learning process proposed by Olsson et al. (2005) and Mallan et al. (2013), it provides more details and specifies when and how the CR to out-groups persists.

Certainly, this account needs to be tested empirically. We think there are three initial ways to do it. First, future studies could examine it using real groups (e.g., racial groups) by adding measures of in-group favoritism and out-group derogation. We predict in-group favoritism would be negatively correlated with the CR to in-groups during acquisition whereas out-group derogation would be positively correlated with the CR to out-groups during both acquisition and extinction. Second, since in-group love is the extension of people's personal self-esteem, it is natural to infer that weaker CR to in-groups during acquisition would occur only for individuals with higher self-esteem. State self-esteem could be manipulated to test this hypothesis. Third, we could also adapt the minimal group paradigm by manipulating the relationship between in-groups and out-groups. Our hypothesis is that if there is a conflict between arbitrarily assigned groups, the conditioning bias, just as what Olsson et al.'s (2005) reported using racial groups (i.e., stronger CR to out-groups during acquisition and remaining CR to out-groups during extinction), would be found.

### Conflict of interest statement

The authors declare that the research was conducted in the absence of any commercial or financial relationships that could be construed as a potential conflict of interest.
